# A Wideband Magnetoresistive Sensor for Monitoring Dynamic Fault Slip in Laboratory Fault Friction Experiments

**DOI:** 10.3390/s17122790

**Published:** 2017-12-02

**Authors:** Brian D. Kilgore

**Affiliations:** U.S. Geological Survey, 345 Middlefield Rd. MS-977, Menlo Park, CA 94025, USA; bkilgore@usgs.gov; Tel.: +1-650-329-4859

**Keywords:** magnetoresistive, linear position sensor, dynamic fault slip, electromagnetic radiation

## Abstract

A non-contact, wideband method of sensing dynamic fault slip in laboratory geophysical experiments employs an inexpensive magnetoresistive sensor, a small neodymium rare earth magnet, and user built application-specific wideband signal conditioning. The magnetoresistive sensor generates a voltage proportional to the changing angles of magnetic flux lines, generated by differential motion or rotation of the near-by magnet, through the sensor. The performance of an array of these sensors compares favorably to other conventional position sensing methods employed at multiple locations along a 2 m long × 0.4 m deep laboratory strike-slip fault. For these magnetoresistive sensors, the lack of resonance signals commonly encountered with cantilever-type position sensor mounting, the wide band response (DC to ≈ 100 kHz) that exceeds the capabilities of many traditional position sensors, and the small space required on the sample, make them attractive options for capturing high speed fault slip measurements in these laboratory experiments. An unanticipated observation of this study is the apparent sensitivity of this sensor to high frequency electomagnetic signals associated with fault rupture and (or) rupture propagation, which may offer new insights into the physics of earthquake faulting.

## 1. Introduction

Many laboratory rock friction experiments have previously focused on steady-state or quasi-static fault motions, related to testing empirical rate- and state-dependent friction constitutive laws [1,2,3,4,5,6,7,8,9] which are widely used to model a variety of earthquake processes. A critical part of laboratory rock friction experiments such as these, is the ability to make detailed and accurate measurements of the fault slip. A variety of position sensing technologies have been employed in laboratory experiments to measure fault slip, including, but not limited to; linear-variable differential transformers (LVDT and DCDT), magnetostriction devices, eddy currents, linear capacitors, foil strain gages, and optical methods. All of these technologies can offer excellent accuracy and resolution performance. Each technology offers advantages and disadvantages with respect to the measurement range, bandwidth, ease of use, signal conditioning requirements, and cost. Experimental conditions also impact sensor selection and performance with respect to mounting and space constraints, line of sight access for optical sensors, and sensitivity to temperature, shock, and vibration.

In steady-state velocity experiments, the shock and vibrations related to stick-slip fault motion are largely absent, and the frequency content of position signals is generally very low, DC to several Hz. In this frequency range, investigators have wide latitude in the selection and mounting of position sensors, as well as in the selection of signal conditioning. While all the previously mentioned methods of measuring fault slip can be used to document total stick-slip fault motion, measurements of the dynamic higher frequency aspects of stick-slip fault motion present challenges to all of these technologies. For example; sensor and signal conditioning bandwidth limitations, limited space near the fault restricting sensor deployment, and shock induced resonance of sensor mounts can degrade position measurements and obscure short period details of the actual fault motion.

In recent years, there has been renewed and increased interest in investigations of the dynamic aspects of fault motion related to earthquakes [10,11,12,13,14,15,16,17,18,19]. Laboratory friction experiments have been employed for many years to study a wide variety of dynamic earthquake processes. The long-standing hypothesis is that laboratory stick-slip friction experiments are essentially earthquake analogs, and have been thoughtfully examined by others, e.g., [11]. Examples of recent investigations into the dynamic motion of laboratory faults include work by Lockner et al. [18] who performed triaxial stick-slip experiments using 25.4 mm diameter × 63.5 mm long cylinder samples of Westerly granite with a saw cut fault that show fault slip rates approaching 20 m/s, and estimates of event durations as short as 70 microseconds. Passelegue et al. [17] also performed triaxial stick-slip experiments using 40 mm diameter × 80 mm long cylinders of Westerly granite with a saw cut fault and report fault slip rates approaching 10 m/s, and event durations measured in 10’s to 100’s of microseconds. Ohnaka et al. [20] previously investigated stick-slip fault motions using 28 cm × 28 cm × 5 and 10 cm thick saw cut samples of Tsukuba granite, and reported peak accelerations measured in 100,000’s m/s^2^, and fault slip velocities approaching 1 m/s. As interest in laboratory stick-slip experiments continues, fault slip sensors used in such tests will need to be rugged wideband instruments in order to accurately resolve the details of rapid dynamic fault motions.

A new method to make accurate and detailed wideband measurements of dynamic fault slip employs a small magnetoresistive sensor, a small but powerful magnet, and custom wide bandwidth signal conditioning electronics. The sensors components are directly attached to the rock samples, eliminating the need for a conventional cantilever type sensor mount. The inability to reposition the sensor or the magnet as fault slip accumulates is accommodated by custom signal conditioning electronics which keep the sensor output on-scale as the experiments are performed and slip accumulates. The analog output of the sensor is sinusoidal, but becomes nearly linear if an appropriate interval of the sensing range is chosen, as in these tests. A calibration technique is presented that converts measured voltage to fault displacement, while maintaining an acceptable level of error. Data generated by the magnetic position sensor in response to stick-slip fault motion along a 2 m long × 0.4 m deep fault are compared to data simultaneously collected by two laser vibrometers measuring the velocity and position (time-integrated velocity) of the sensor and magnet, and other nearby position and strain sensors to demonstrate the capabilities, and sensitivity of this position measuring method.

An unanticipated observation of this study is the apparent sensitivity of this sensor to electromagnetic (EM) signals generated during dynamic stick-slip along the 2-m fault used in these tests. While clear EM signals have long been observed nearby, and at the time of many large earthquakes and volcanic eruptions apparently related to crustal stress release, no unambiguous observations of EM precursor behavior in the epicentral region have been observed [21,22,23,24]. Some associations of global EM disturbances in the ionosphere and magnetosphere prior to large earthquakes have been made [25], but these observations appear to result from inadequate correction of normal EM disturbances and selective use of data only prior to these earthquakes [26,27,28,29,30,31,32,33,34,35]. In addition to field studies, numerous laboratory investigations have documented EM emissions related to the fracture of rock samples [36,37,38,39,40,41,42,43,44,45,46]. A smaller number of laboratory investigations have investigated EM emissions related to rock friction and stick-slip fault motions, generally using relatively small samples and single point measurements of EM radiation [47,48,49,50,51]. No experiments appear to have used an array of sensors along a sufficiently long fault to observe EM emissions related to propagating fault rupture processes, as this report documents. Nevertheless, the search for the physics behind EM emissions related to earthquakes and volcanic processes remains the subject of debate [52].

Various physical mechanisms have been identified as contributing to the generation of earthquake and volcanic related EM emissions. These include piezomagnetic effects, electrokinetic effects, induced EM signals from seismic wave motion, triboelectric effects and other charge generation processes [22]. Piezomagnetism is the property of some ferro-crystalline materials where induced and remnant magnetism changes with applied stress. Electrokinetic effect can result from tectonic stress induced flow of conductive fluids through porous, permeable and conductive rock mass [53]. EM signals can also be generated by motion of the earth’s crust within the dynamic and static geomagnetic field, caused by crustal loading and/or seismic waves [54]. EM effects can be both fast and slow since movement of the crust can occur fast (earthquakes) and slowly (tectonic loading). Triboelectric effects result from charge separation during microcracking and rock shearing around the rupture surface [23]. Regardless of the origin of the signals detected in this report, which are not known, the study of the origin and quantification of suspected EM signals observed in these tests requires equipment and techniques beyond the scope of this report. Accordingly, the discussion of the physical origin of EM signals observed here will be the subject of further investigation.

## 2. Materials and Methods

This measurement technique relies on the Honeywell HMC1501 [55,56,57], a high resolution anisotropic magnetoresistive linear, angular, and rotary displacement sensor in a Small Outline Integrated Circuit (SOIC) surface-mount package, referred hence forth as the AMR sensor. Magnetoresistance is a property of some ferrous materials where electrical resistance varies as a function of the strength of an applied magnetic field. Anisotropic magnetoresistance (AM) is a property of some magnetoresistive materials, where the electrical resistance of the material varies as a function of both the strength and direction of an externally applied magnetic field, as well as the path of current flow through that material [58,59,60,61]. The magnitude of the change in electrical resistance of Permalloy, the AM material used in these AMR sensors, in response to changes in the orientation of an applied magnetic field, is as much as 2 to 3 percent at room temperature. For comparison, a standard 350-ohm foil strain gage with a gage factor of 2, subject to 1000 micro-strain of shortening or elongation, will change the resistance of that gage by only about 0.2 percent. A sufficiently strong, or ‘saturating’ magnetic field maximizes the AM effect in magnetoresistive materials while simultaneously minimizing any effects of stray external magnetic fields. Once the magnitude of the applied magnetic field exceeds the saturation threshold of the AM material, and the path of current flow through the AM material is fixed, the electrical resistance of that AM material then should only vary with the direction of the applied magnetic field, which is the principle behind the functionality of this AMR sensor.

This AMR sensor is considered ‘saturated’ if used in a magnetic field with a strength equal or greater than 80 oersteds (approximately 80 gauss) [57]. For reference, the intensity of the Earth’s magnetic field, ***F***, in Menlo Park, CA is approximately 50,000 nT or 0.5 Gauss [62]. A small rare-earth neodymium magnet with a nominal surface field strength of 5876 Gauss, item number B824 from K&J Magnetics, Inc. (Pipersville, PA, USA) [63], was identified as one of many suitable magnets for use with the AMR sensor for these tests. The strength of the magnetic field around the AMR sensor, with a 5-mm gap between the magnet and the sensor, as deployed in these tests, was found to exceed 80 Gauss using a model GM1-ST DC Gauss meter from AlphaLab Inc. (Salt Lake City, UT, USA) [64].

Identifying magnets with sufficient field strength to ‘saturate’ the AMR sensor is a straight forward process. Identifying the shape of the magnetic flux planes that emanate from a magnet, and evaluating the geometry of how the sensor will cut through those flux planes during anticipated slip motion is a more complex process and must be considered when selecting a magnet to pair with the sensor. The AMR sensing bridge is a two-dimensional structure, while magnetic field flux can vary in three-dimensions. One method to identify the orientation of the flux planes of a target magnet to aid in the positioning and planned motion of the sensor and its target magnet is to use a 3-axis (*X*-*Y*-*Z*) linear translation stage to precisely move the AMR sensor and magnet relative to each other, generate contour plots of equal signal output from the AMR sensor, and map the flux planes of the target magnet. The AMR sensor generates the largest change in the analog signal output when it and its magnet are positioned such that the relative motion between the sensor and the magnet causes the greatest number of magnetic field lines to orthogonally cross the sensing element of the AMR sensor. Careful consideration must be given to how undesirable or unexpected motions of either the sensor or the magnet may influence the signal output of the sensor. The AMR sensor has a continuously varying analog voltage output and is capable of resolving angles between planes of magnetic flux as small as 0.07 degrees. The signal to noise of the signal conditioning used in these tests impacts sensor resolution as well. As deployed in these tests, with a 5-mm gap between the sensor and a laterally moving magnet, 0.07 degrees of angular resolution translates into a fault slip resolution of approximately 6 microns. For these tests sensor response was examined for both the anticipated fault parallel/horizontal fault slip motion to develop calibration curves for the slip displacement data, as well as for possible differential vertical fault motions, to determine the transverse sensitivity of the AMR sensor.

**Figure 1:**
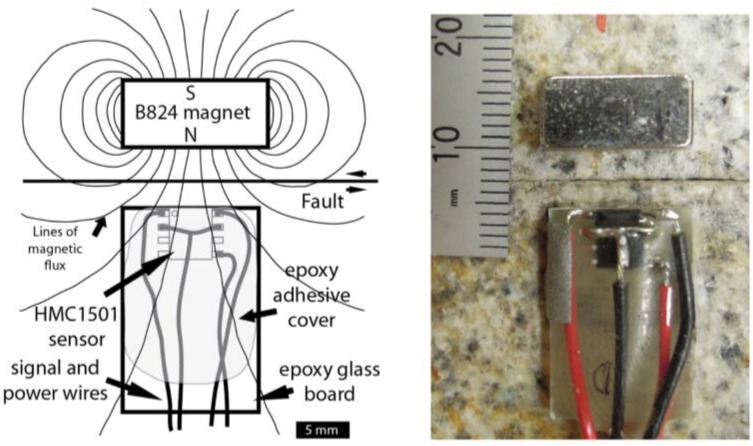
Diagram, left, and photo, right, of the Honeywell HMC1501 magnetoresistive position sensor used in this study. The red, black, and uninsulated wires in the photo are under a cover of clear epoxy. The gray vertical strip partially covering the left most red wire in the photo is retroreflective tape which is the target material for the laser vibrometers to determine the velocity of the sensor. Another piece of retroreflective tape, not seen, is on the right side of the magnet.

To facilitate the use of the AMR sensor, the SOIC chip was first attached to a small (19 mm × 13 mm × 1.6 mm) piece of solid epoxy glass composite prototyping board (no copper cladding or pre-drilled holes) using a slightly viscous cyanoacrylate adhesive (Loctite 454). The prototype board is a rigid inert (electrically non-conductive, and non-magnetic) platform for the SOIC chip, and greatly facilitates the subsequent handling, wiring, and installation of the sensor assembly. Shielded twisted pair wiring is used to make the power-in and signal-out connections with the sensor. A top coat of two-part epoxy is used to encapsulate and firmly secure the SOIC chip and the attached lead wires to the prototype board. The result is a low profile, rugged, easy to handle sensor assembly. When deployed, the magnet and sensor assembly are bonded directly to the test samples using the same cyanoacrylate adhesive, eliminating the need for additional sensor mounting fixtures (Figure 1).

Since the sensor components are permanently attached to the samples and not physically adjustable, the signal conditioning circuit needs to electronically compensate for continuously accumulating fault slip over many stick-slip events that would otherwise cause the sensors amplified signal output to shift off scale. Sufficient amplification of the sensor signal is also required so details of quasi-static and dynamic sensor motion are resolvable. The signal conditioning electronics are comprised of a reference voltage source to provide a stable excitation voltage to the sensor, a two-stage amplifier with a user adjustable output offset between the two amplifier stages, and low-pass active filters for the signal outputs as needed. For details regarding the signal conditioning for the AMR sensor as used in this study, see the Appendix in this article.

**Figure 2:**
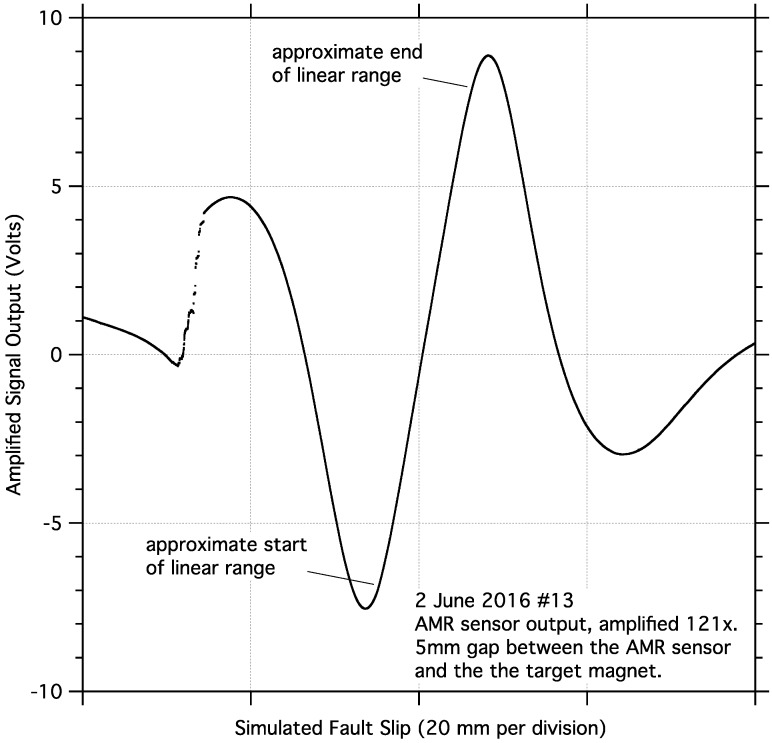
Example of the amplified output of the AMR sensor demonstrating its approximately sinusoidal output over an extended range of linear motion. The output of the AMR sensor is nearly linear over a more limited range of linear motion, which is exploited in these tests.

**Figure 3:**
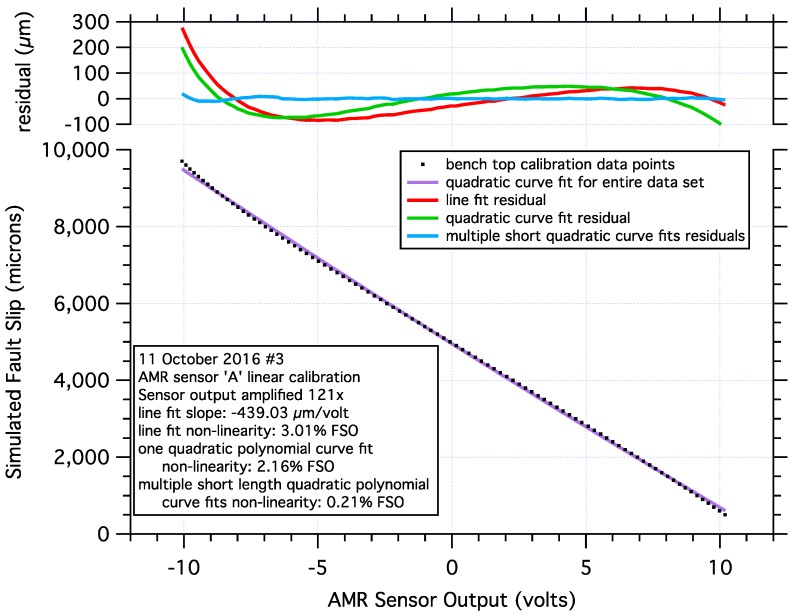
Example of desktop linear calibration data used in these tests showing the nearly linear output of the AMR sensor in response to nearly 10 mm of simulated fault motion. A quadratic curve fit, along with the residuals for; a line fit, a quadratic curve fit for the entire data set, and multiple short length quadratic curve fits of short segments of the calibration data. Short ≈ 1.5 mm) segments of the calibration data are fit with polynomial curves to scale the data collected in these tests. The polynomial curve fits reduce maximum residual errors from 100’s of microns to 10’s of microns resulting in more accurately scaled AMR position data.

The analog signal output of the AMR sensor, responding to relative motion of the sensor and a magnet, is approximately sinusoidal, Figure 2. However, within a limited range of motion, a significant portion of the sensor output is approximately linear, and exploited to measure fault slip in these tests. The desktop calibration of the sensor was performed using a precision linear translation stage(s) paired with manual micrometer head with 20 micron graduations and 1 micron of sensitivity. The sensor and magnet were attached to the calibration stand using plastic mounts to maintain a minimum gap of about 4 cm between the sensor/magnet and any metal to minimize any unwanted magnetic interactions. The 5-mm gap separating, and the relative motion of the sensor and magnet in the calibration stand, are both exactly as they are deployed on the sample blocks. Calibration data were collected from the signal output of the first (lower gain) stage of the signal conditioning circuit at 100 micron intervals. Representative calibration data with a quadratic curve fit are shown in Figure 3. For additional details regarding the calibration of this sensor, see the Appendix in this article.

Determining a static position calibration for a position sensor is a straight forward procedure. Directly determining the frequency dependent response of a position sensor over a range of frequencies likely to be encountered during dynamic stick-slip motion poses more difficulties. The problem is the lack of a vibration source, calibrated or not, that can vibrate a sensor or sensor target, at frequencies from Hz to several 10’s of kHz, with motion of sufficient amplitude that the sensor can accurately resolve. Portable commercial reference shakers typically operate at 1000 rads/s (159.2 Hz) with 10 microns (rms) of motion, though some reference shakers have peak frequencies as high as 1 kHz to 10 kHz. Costlier laboratory benchtop piezo vibrators can generate vibrations at frequencies of several 10’s of kHz. However, as the frequency of the vibration increases from Hz to 10’s of kHz, the amplitude of the motion of the reference shaker necessarily decreases from microns to nanometers. The limited range of motion of high frequency reference shakers is likely at or below the resolution or noise threshold of most position sensors, thus preventing their use in any sort of meaningful calibration procedure. In these tests, spectral analysis of the sensor signals is used to evaluate their frequency response.

The dynamic performance of the AMR sensor used in this study was verified directly using a pair of Polytec CLV-2534 laser vibrometers, which use heterodyne interferometry techniques to generate wide bandwidth high resolution measurements of velocity, which can later be integrated to position for direct comparison to the AMR sensor measurements. The vibrometers are attached to a rigid scaffold above the sample blocks, and the scaffold is resting on the floor of the lab. All vibrometer measurements are therefore referenced to the floor of the lab. Ninety-degree beam turners attached to the vibrometers direct their laser beams to their respective targets so the beams are orthogonal to the target surfaces, and parallel with their anticipated motion. The vibrometers separately measure the velocity of the sensor and magnets on opposite sides of the fault, and those signals are summed or differenced (depending on the relative position of the vibrometer and motion of the target) to produce differential fault slip velocity which is integrated to a fault slip displacement signal. Scaled velocity signals from the vibrometers are linearly de-trended to remove any subtle instrument drift, which improves the quality of position signals integrated from the velocity signals. Fault slip displacement determined by the laser vibrometers should in principle, exactly match the fault slip determined by the AMR sensor. In contrast, the AMR sensors measure fault slip displacement directly across the fault. Vibrometer #1 performance specs: 0 to 350 kHz frequency response, and a frequency dependent resolution of typically 0.06 (µm/s)/√Hz. Vibrometer #2 performance specs: 0.5 Hz to 5 MHz frequency response, and a frequency dependent resolution, typically 0.5 (µm/s)/√Hz.

**Figure 4:**
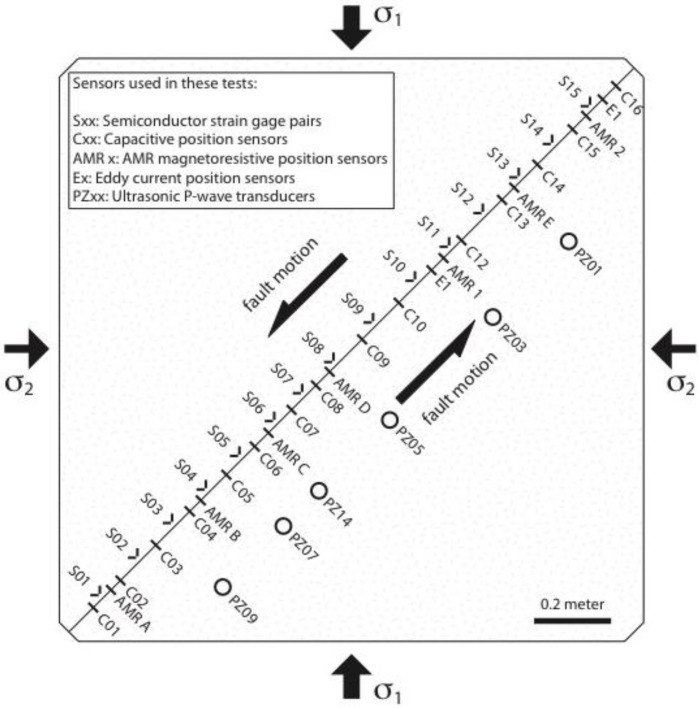
Diagram of the two Sierra White granite sample blocks as they are used in the USGS biaxial test apparatus in Menlo Park, CA, viewed from above. The locations of the fault slip, shear stress, and ultrasonic sensors used in these tests are positioned to scale. The diagonal line from lower left to upper right where the two granite samples meet is the simulated strike-slip fault. The fault plane is approximately 2.05 m long and 0.4 m deep.

Stick-slip experiments were performed using the large bi-axial test apparatus, using Sierra White granite samples with a simulated strike-slip fault, 2-m-long and 0.4 m deep, located at the U.S. Geological Survey in Menlo Park, CA, USA [10,12,13,65,66,67,68,69] (Figure 4). A constant normal stress of 5 MPa was imposed on the fault, and a constant shear stress loading rate of 1 kPa/s was applied to the fault until a dynamic stick-slip event was generated. After the initial shear stress loading and stick-slip fault motion, the loading process was repeated several more times until subsequent loading cycles produced nominally consistent events, from which the data in this report were obtained. The initial shear stress for each subsequent loading cycle was the residual shear stress of the preceding event. The time between subsequent shear loading cycles was neither systematic nor considered in the analysis of these data. Peak shear stress immediately prior to stick-slip was approximately 3.9 MPa, with a stress drop of approximately 0.4 MPa for most stick-slip events in this study. During stick-slip, all sensor signals were simultaneously recorded at either 1 MS/s (12-bit resolution), 1 MS/s (16-bit resolution), or 10 MS/s (14-bit resolution) for a fraction of a second using three 50%/50% pre-trigger/post-trigger recording systems. The 10 MS/s data were later resampled to 1 MS/s to facilitate the merger of all of the data sets. All experiments were conducted at room temperature under dry conditions.

**Figure 5:**
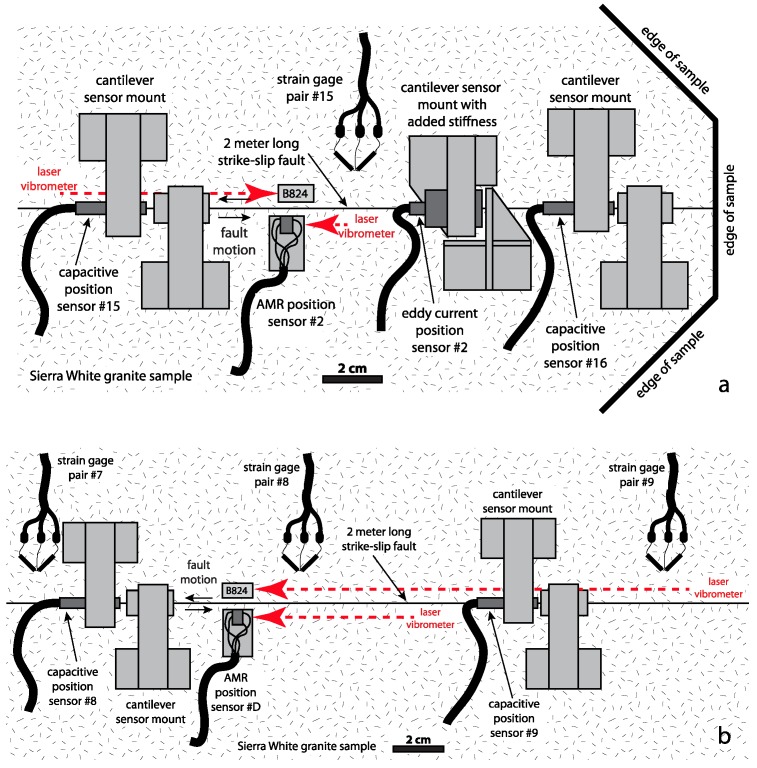
Diagram to scale showing the location of the AMR sensor and other close by sensors used in this report near one end of the 2-m fault (**a**), and near the middle of the fault (**b**). The red dashed lines with arrows show the origins and paths of the laser vibrometer laser beams separately, but simultaneously measuring the velocities of these AMR sensor/magnet pairs.

Fault slip data generated by the AMR sensor array were compared to fault slip data simultaneously generated by a variety of other slip, strain, and ultrasonic sensors deployed along the length of the 2-m fault (Figure 4). The performance of the AMR sensors was compared directly to laser vibrometer measurements at locations near the middle and at the end of the 2-m fault (Figure 5). An array of 15 semiconductor strain gage pairs, configured to measure shear strain/stress, are deployed along the length of the 2-m fault. The frequency response of the strain gage pairs and their signal conditioning used in these tests is DC to ≈ 100 kHz. An array of 15 Capacitec HPC-40 position sensors paired with 4100-SL signal conditioners (specified frequency response: DC to 3.1 kHz) mounted in simple cantilever style mounts, are also deployed along the length of the 2-m fault to measure fault slip. Two Micro-Epsilon U1 position sensors paired with eddyNCDT 3010 signal conditioners (specified frequency response: DC to 50 kHz) were also deployed along the 2-m fault where space permitted. The standard response of the Micro-Epsilon sensor is DC to 25 kHz, the response reported here is the result of a factory modification to the eddyNCDT 3010 hardware. The mounts used with the eddy current sensors are designed to mitigate the resonance vibrations previously observed in the capacitive sensor records. Signals captured by an array of five 1 MHz Panametrics V103-RB ultrasonic P-wave transducers with a -6 dB passband between approximately 450 kHz and 1.6 MHz and a 13 mm nominal element diameter, were also employed in the analysis of the signals generated by the AMR sensor. The ultrasonic transducers were previously deployed to capture high frequency acoustic signals related to earthquake nucleation and rupture processes [12]. While all of the fault slip, fault velocity, and shear stress sensors are positioned within several cm of the fault trace to monitor motions along the surface fault trace, the ultrasonic transducers are positioned 20 cm from the fault trace, one half the depth of the fault, to monitor AE emanating from the entire fault plane.

Lacking specialized equipment to properly document the unanticipated, but presumed EM signals observed in these experiments, a test was devised using available equipment. One HMC1501 SOIC chip and a target magnet were glued to a single piece of epoxy glass composite prototyping board with the same 5-mm gap as if deployed on the granite samples, and employed one of the existing signal conditioning modules. This sensor was deployed at several locations along the 2-m fault, both simply resting on the sample block spanning the fault, and later, still spanning the fault, but suspended a few millimeters above the sample block attached to the same scaffold supporting the laser vibrometers. The output of this test AMR sensor should be constant since the sensor/magnet pair are attached to the same piece of epoxy fiber board and should not experience any differential motion, and that sensor assembly is either lightly resting on or simply not physically connected at all to the granite sample blocks to minimize or eliminate any signals generated by any motion or vibrations of the sample blocks or test apparatus.

## 3. Results

### 3.1. AMR Sensor Calibration

Calibration data from the AMR sensors were obtained from five separate sensor/magnet pairs. Each sensor-magnet pair was separated by a 5-mm gap, as shown in Figure 1, and total lateral displacement of the magnet with respect to the sensor was about 9.2 mm, simulating cumulative maximum fault slip on the 2-m fault in the biaxial test apparatus. The 9.2 mm of simulated fault slip generated an output signal of approximately ±10 volts via the signal conditioning amplifiers low gain (200×) output. Calibration data acquired at 100 micron intervals produced detailed records to which a variety of scaling methods can be applied. See the Appendix for details of applying low gain calibrations to high gain recorded signals. Simple line fits of the position data over the full range of the approximately linear output range of the sensor (Figure 3), produced fits with maximum non-linearity errors that varied from 2.4% to 3.2% of the full-scale output. The central (≈ 4.3 mm displacement and ≈ ±5 volts output) portion of the calibration curves produced line fits with maximum non-linearity errors that varied from 0.16% to 0.36%. A second degree, or quadratic polynomial curve fit over the same 9.2 mm calibration span produced curve fits with maximum residual errors between 1.2% and 2.3% of the full-scale output. Quadratic polynomial curve fits over shorter, ≈1.5 mm portions of the full 9.2 mm span, produced curve fits with maximum residual errors as high as 0.88% of the full-scale output at the ends of the 9.2 mm span, and maximum residual errors as low as 0.06% towards the middle of the 9.2 mm span. Higher order polynomial and other curve fits could also be applied to these data. For this analysis, simple quadratic curve fits of short segments of the calibration curves were used to scale the recorded sensor signals to fault slip displacement data.

Calibrations were also performed to determine the sensitivity of the deployed sensors to vertical cross fault motions that may accompany dynamic fault slip that could register as fault slip by the AMR sensors. A 3-axis linear translation stage was used examine the sensitivity to simulated differential vertical fault motions of ±0.4 mm at 1 mm intervals along the 9.2 mm roughly linear output range of the AMR sensor. The transverse sensitivity of the AMR sensor to vertical motion as deployed in these tests is estimated to vary from as 0.4% to as high as 3.2%, with an average of 1.8% ±1.0%. Using the average transverse sensitivity of 1.8%, a differential vertical fault motion of about 56 microns would generate about 1 micron of apparent fault slip displacement. In tests using the laser vibrometers to measure differential vertical fault motion at several locations along the 2-m fault, maximum differential vertical fault motion of just a few microns was observed. In these tests, apparent fault slip displacement resulting from low amplitude vertical fault motions would likely be obscured by the background noise of the AMR sensor signals.

The signal conditioning circuit frequency response was determined to be −3 dB at about 700 kHz. Since the specifications of the AMR sensor lists a bandwidth of 5 MHz, the currently used signal conditioning circuit limits the overall bandwidth of the sensor system. The frequency of the signal conditioning electronics was determined by using a function generator to supply a small amplitude sine wave signal to the amplifier input, and using an oscilloscope to monitor the attenuation of the amplitude signal (±10 volts nominal) as the source frequency varied. The high bandwidth capability of the signal conditioning circuit likely contributes to noise in the recorded signals, a 15.63 kHz small amplitude wave, present in each of the AMR sensors signals. The use of a 200 Hz wide notch filter during post-processing of the data appears to reduce that noise signal to acceptable levels without degrading the data.

The sensitivity of the AMR sensor SOIC chip to temperature changes is well documented by the manufacturer [57]. A quick and simple test of the sensitivity of the AMR sensor to temperature changes as deployed in these tests was performed using a hot air gun and a K-type thermocouple. The hot air gun was used to raise the temperature of a deployed AMR sensor #2 approximately 10 °C above the ambient room temperature, while the amplified voltage output of the sensor was monitored. A small quantity of heat conducting paste on the top surface of the AMR sensor was used to facilitate the thermal contact between the thermocouple and the AMR sensor. Over this limited range of temperature change, the AMR sensor appears to show a sensitivity of about 2 microns of apparent fault slip per degree C of temperature change.

### 3.2. Dynamic Fault Slip Measurements

**Figure 6:**
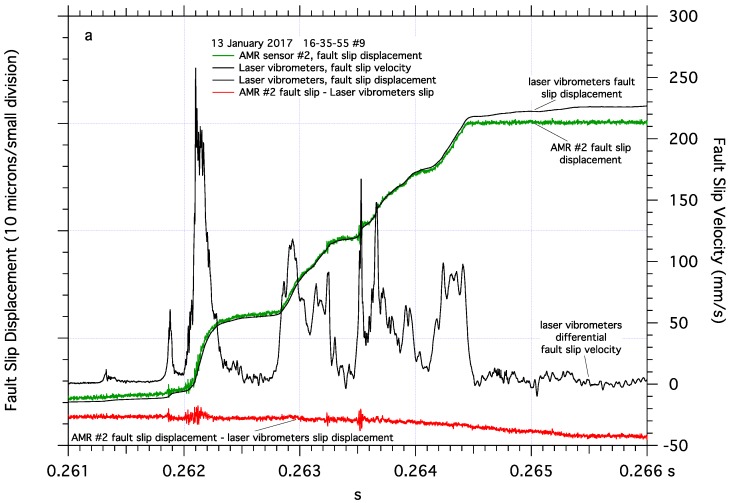

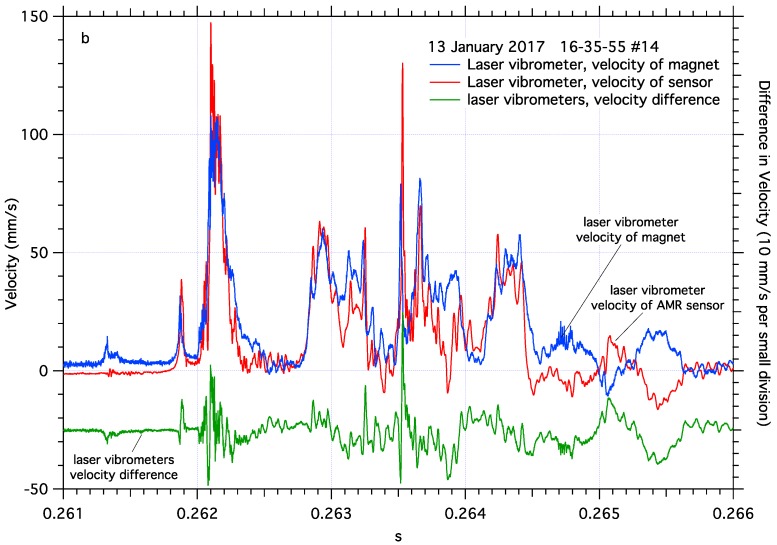
Detail of dynamic fault slip near one end of the 2-m fault revealing a complex pattern of episodic slip. Differential fault slip velocity and comparison between fault slip measured directly by AMR fault slip sensor #2 and fault slip determined by integrating differential fault slip velocity from laser vibrometer measurements. Short periods of higher frequency oscillations in the AMR fault slip record are easily identified when the AMR fault slip record is compared to the laser vibrometer fault slip record (**a**). Details of the two laser vibrometer measurements, one tracking the AMR #2 sensor and the other tracking the motion of its target magnet across the fault, and the difference between those two signals (**b**).

The performance of the AMR sensor measuring dynamic fault slip is determined at several locations along the 2-m fault by comparing fault displacement measured directly by the AMR sensor/magnet pair glued to the samples, and the differential displacement (time integrated velocity) of the same AMR sensor/magnet pair simultaneously determined by the laser vibrometers. The fault slip displacement obtained from AMR sensor #2 mounted near one end of the 2-m fault compares favorably to the fault slip determined by the laser vibrometers (Figure 6a). Dynamic fault slip at the end of the fault is complex and characterized by an impulsive onset of fault motion followed by a series of short episodes of rapid slip over a background of slower slip. The slip record from the AMR sensor clearly tracks the episodic slip, and reveals bursts of higher frequency signal throughout the slip event. The simultaneously acquired differential velocity of the AMR sensor/magnet pair integrated to fault slip displacement correlates closely with the fault slip displacement measured directly by the AMR sensor, though the vibrometer data does reveal additional slip after dynamic slip ends at 0.2645 s, and does not appear to detect the higher frequency oscillations detected by the AMR sensor. Subtracting the laser vibrometer displacement record from the AMR slip record reveals the distribution and magnitude of the higher frequency signals detected by the AMR sensor, as well as the post dynamic slip fault motion.

A closer examination of the vibrometer velocity data for the AMR sensor and its magnet (Figure 6b) shows approximately symmetrical velocities across the fault as expected. However, near the end of the dynamic slip, around 0.2645 s, the previously tracking velocity signals become out of phase which is coincident with the separation of the AMR sensor fault slip record and the laser vibrometer fault slip record. Differencing those two vibrometer records reveals the asymmetry of sample block slip velocity near the end of the fault, with peak to peak differences in velocity across the fault approaching 100 mm/s. Much of the difference between the two velocity records appears as short period variations in relative block velocities, likely representing very small changes in fault displacement. However, as dynamic slip ends, the period of the velocity difference signal increases, and could account for the additional apparent fault slip following the dynamic fault slip, detected by the laser vibrometers.

**Figure 7:**
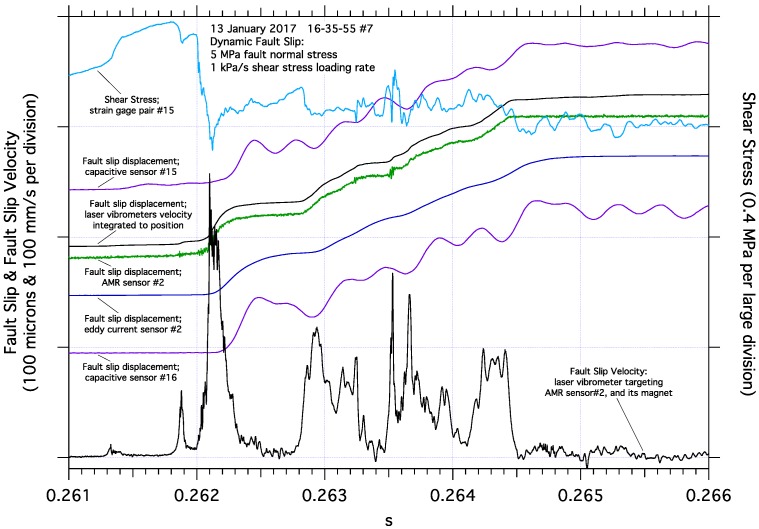
Details of dynamic fault slip near one end of the fault including nearby fault slip, shear strain, and laser vibrometer measurements. In this example, fault slip accumulates via a series of episodic impulsive slip events, clearly illustrated by the AMR and laser vibrometer fault slip signals, and to a lesser degree by the eddy current fault slip sensor. The oscillations in the capacitive fault slip sensor signals reflect those sensor mounts resonating at approximately 3 kHz. The shear stress record reveals an abrupt drop in shear stress as dynamic slip initiates.

The magnitude of fault slip displacement at the end of the fault determined by the AMR sensor also compares favorably to the fault slip displacement data from both the capacitive and eddy current position sensors (Figure 7). The eddy current signal tracks the episodic slip and total slip, though with what appears to be slower rise times compared to either the AMR sensor or the capacitive position sensors. The cause for what may be a sluggish response of that sensor is unknown, though may be related to nylon components incorporated into the eddy current sensor mount and requiring diagnostics beyond the scope of this report. Neither the capacitive nor the eddy current position sensors appear to detect the high frequency signals seen in the AMR record. The capacitive sensors should not be capable of detecting motion at frequencies much beyond 3.1 kHz, but the eddy current sensors should be responsive to motion at frequencies as high as about 50 kHz. Figure 7 also illustrates the unfortunate effects of sensor mount resonance seen in both capacitive slip sensor records, though both capacitive sensors do accurately capture the total amount of slip during the event. The sensor mounts currently used with the capacitive position sensors have a measured resonance frequency of about 3.2 kHz which is consistent with observed oscillations in those slip records. The eddy current fault slip sensor mount also employs a cantilever design, however, it has stiffening elements that appear to mitigate negative resonance effects. The strain gage pair, measuring local shear stress on the fault, shows a complex relationship with the close by slip and velocity records. The abrupt increase in fault slip rate as slip initiates at the end of the fault appears to correlate with an abrupt drop shear stress at the end of the fault where strain pair 15 is located. Neither the episodic fault slip nor the bursts of high frequency signal seen in AMR fault slip #2 record are readily apparent in the shear stress record from strain gage pair #15.

**Figure 8:**
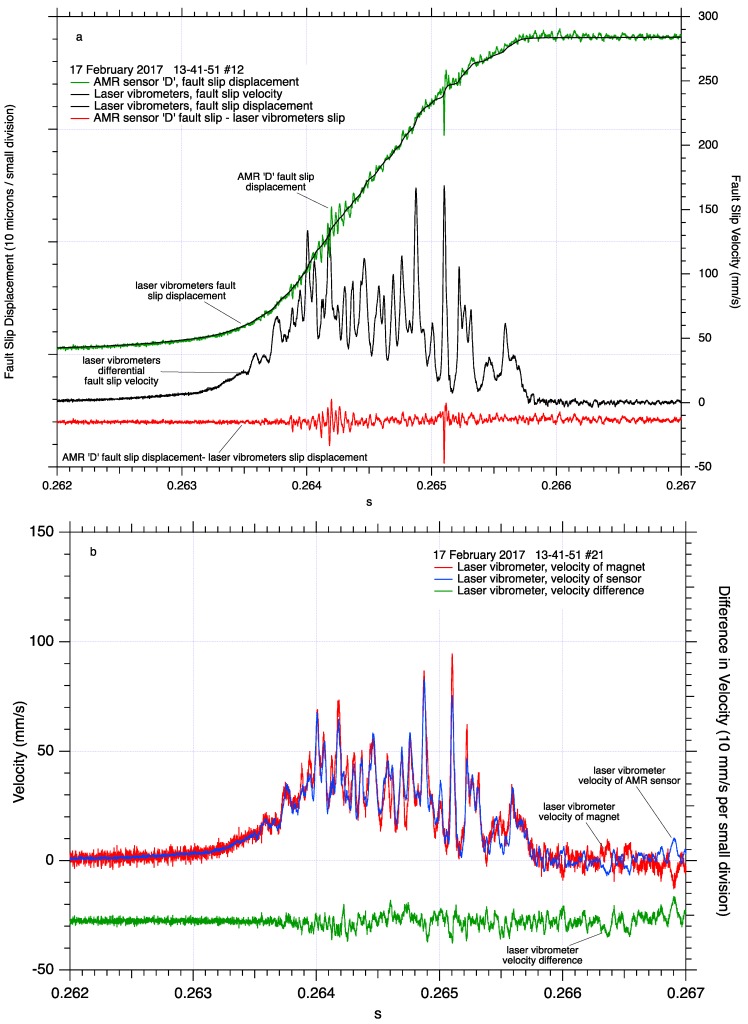
Detail of dynamic fault slip near the middle of the 2-m fault revealing a less complex pattern of continuous fault slip. Differential fault slip velocity and comparison between fault slip measured directly by AMR fault slip sensor #D and fault slip determined by integrating differential fault slip velocity from laser vibrometer measurements. Short periods of higher frequency oscillations in the AMR fault slip record are easily identified when the AMR fault slip record is compared to the laser vibrometer fault slip record (**a**). Details of the two laser vibrometer measurements, one tracking the AMR #D sensor and the other tracking the motion of its target magnet across the fault, and the difference between those two signals (**b**).

In contrast, the initiation of dynamic fault slip near the middle of the fault measured by AMR sensor ‘D’, Figure 8a, is emergent, with higher frequency oscillations superimposed in both the AMR slip displacement and the differenced laser vibrometer velocity records. Fault slip displacement determined by the laser vibrometers, is an excellent match to fault slip displacement determined directly by the AMR sensor. Consistent with measurements shown in Figure 8, fault slip displacement determined by the vibrometers at this location also appears to lack the high frequency content detected by the AMR position sensors. Subtracting the laser vibrometer slip record from the AMR slip record highlights the higher frequency signals the AMR sensor detected, though with no additional post dynamic slip fault motion detected by vibrometer measurements at the end of the fault, (Figure 6a). Examination of the individual velocity records, monitoring the motion of AMR sensor ‘D’ and its target magnet, Figure 8b, shows synchronous velocity records for the sensor and the magnet during and following dynamic slip, in contrast to similar measurements near the end of the fault, Figure 6b. The difference between the two velocity signals near the middle of the fault show short period peak to peak differences between the two velocity records to be less than 50 mm/s, and no apparent fault slip following dynamic fault slip after 0.2658 s.

**Figure 9:**
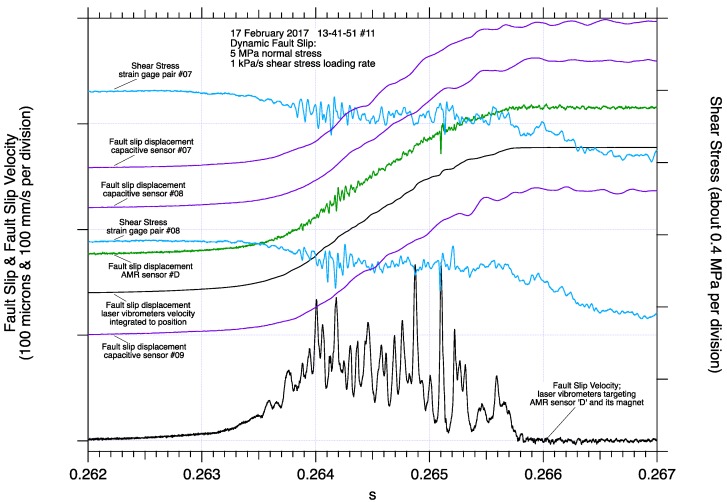
Details of dynamic fault slip near the middle of the fault including nearby fault slip, shear strain, and laser vibrometer measurements. The lack of an impulsive onset of fault slip near the center of the fault is reflected in all of the slip, stress and velocity records. The lack of resonance in the capacitive slip sensor records is additional evidence of the gradual evolution of fault slip accumulation near the center of the fault. Periods of higher frequency oscillations in the AMR fault slip record stand out, and appear to correlate with higher frequency oscillations observed in the shear stress records.

Slip records from AMR sensor ‘D’, as well as close by capacitive and eddy current position sensors (Figure 9), closely track each other in both shape and magnitude. The lack of an impulsive onset of dynamic slip at this location likely inhibits resonance of the capacitive sensor mounts improving the quality records of those fault slip displacement records. Strain gage pairs #07 and #08 located on either side of AMR sensor D both show a gradual decline in fault shear stress as fault slip accumulates consistent with observations of the gradual evolution of fault slip and fault slip velocity at that location along the fault. The higher frequency oscillations seen in the AMR sensor fault slip displacement record may correlate to high frequency oscillations seen on both strain gage pair records.

The time-series presentation of laboratory stick-slip data is especially helpful for determining earthquake source parameters analogous to those observed seismically including; rise-time and event duration, peak and average fault slip rates, the identification of rupture propagation speeds, and the relative timing of specific details of slip, stress, acoustic emission, etc. However, other earthquake source parameters are best identified and (or) visualized when laboratory stick-slip data are referenced to wide-band measurements fault slip displacement. Some of those parameters include; fault unloading stiffness and machine unloading stiffness, the slip weakening or critical slip distance, peak stress, average stress, final stress, apparent stress, overshoot, and the graphical representation of the energy budget of an earthquake (friction energy, fracture energy, and radiated energy), etc. An analysis of these parameters is beyond the scope of this report, however, for more information about how these parameters are relevant to earthquake processes, see [1,2,3,4,5,6,7,8,9,10,11].

**Figure 10:**
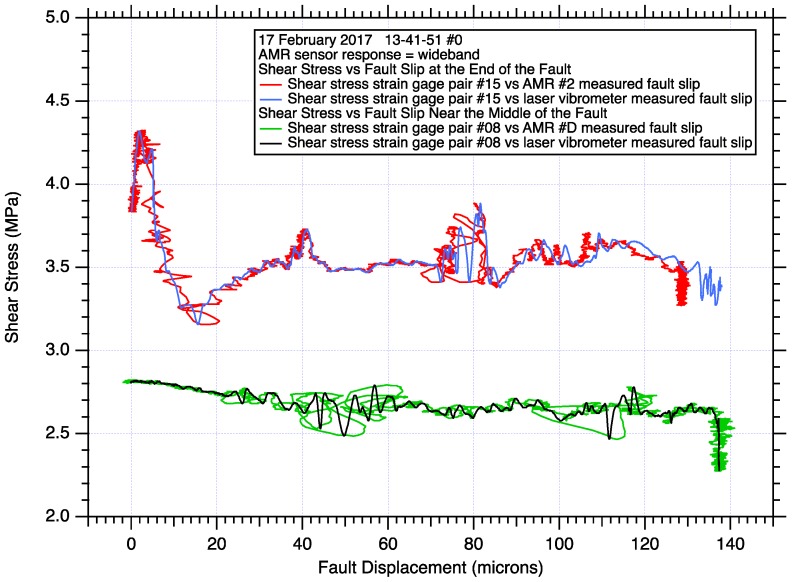
Fault shear stress during stick-slip fault motion derived from strain gage pair records located at the end of the fault and near the middle of the fault, plotted against fault slip displacement determined by both the closest AMR fault slip position sensor (about 2.5 cm separation in both cases, see Figure 5), and by the laser vibrometer pair tracking those same AMR sensors and their matching magnets.

Plots of shear stress vs measured fault slip (Figure 10), from data collected at the end of the fault and near the middle of the fault, illustrate some of the slip dependent characteristics of stick-slip fault motion. The upper curves show the abrupt initiation of stick-slip fault motion at the end of the fault. The end of the fault has remained locked, no fault slip, throughout most of the earthquake nucleation process. Rapid stick-slip fault motion initiates along the fault when the accelerating stable slip in the expanding nucleation zone reaches the end of the fault. The peak stress, the critical slip displacement (measuring less than 20 microns), and high frequency oscillations in both the shear stress and the AMR fault slip records, are all readily apparent. In contrast, the lower curves show shear stress vs. fault slip near the middle of the fault. The nucleation process has been active near the middle of the fault for some time prior to the initiation of stick-slip fault motion. During the nucleation process, the middle section of the fault has experienced stable accelerating fault slip which causes the local shear stress to gradually reduce. When rapid stick-slip fault motion reaches the center portion of the fault, the transition in fault slip velocity is gradual (Figure 9) compared to the abrupt fault slip velocity transition at the end of the fault (Figure 7).

When comparing the curves of shear stress vs. fault slip in Figure 10, the reference measurements from the laser vibrometers produce a superior low-noise wide-band fault slip displacement record which allows the details of the shear stress records, including high frequency oscillations, to be easily identified. In contrast, the wide-band AMR fault slip displacement records contain several microns of background noise which obscure some record details. The AMR fault slip records also contain high frequency oscillations themselves (Figure 6, Figure 7, Figure 8 and Figure 9) that are coincident with oscillations in the shear stress records, resulting in loops in the data plots. Low pass filtering and other processes can be applied to the AMR fault slip data to reduce noise, though at the possible expense of higher frequency signal content. Signal filtering decisions are likely to be made on a case-by-case basis with the aid of spectral analysis of the data and a review of the goals of the project.

**Figure 11:**
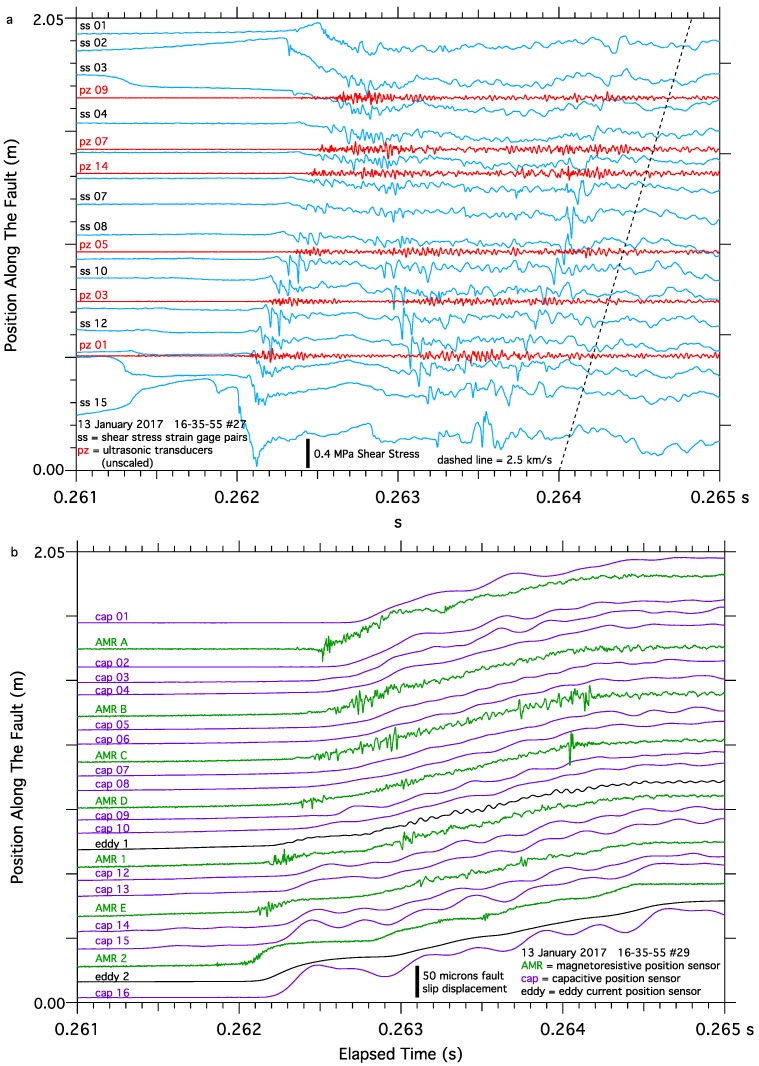
Data collected from all the sensors deployed along the 2-m fault during one stick-slip event. Each signal trace is plotted so that its position along the y-axis approximately corresponds to that sensors location along the fault trace to facilitate spatial and temporal comparisons. Shear stress and acoustic emissions, (**a**), show fault rupture initiate at about 0.262 s near strain gage #15 and propagate (manifested as higher frequency oscillations in the sensor records) to strain gage #01. Fault rupture also appears to initiate near strain gage #01 about 300 micro-seconds later, resulting in a complex pattern of rupture propagation and reflections along the length of the fault. Fault slip signals, (**b**), capture rupture initiation near capacitive fault slip sensor #16 and propagating to capacitive fault slip sensor #01. Fault rupture propagation appears to be detected by the AMR fault slip sensors as higher frequency oscillations in the signals.

To put the data collected in the immediate vicinity of the AMR sensors into perspective, data collected along the entire length of the 2-m fault from all the fault slip and shear strain sensors, as well as the acoustic emission signals from the ultrasonic transducers, are shown in Figure 11a,b, separated into two plots for clarity. In this test apparatus, the nucleation process typically begins near the center of the fault many 10’s to 1,000’s of seconds preceding earthquake initiation determined by the shear stress loading rate imposed on the fault. As the earthquake initiation approaches, fault creep accelerates near the center of the fault and shear stress simultaneously degrades. Eventually the width of the nucleation zone expands to the length of the fault. Dynamic fault rupture initiates at approximately 0.262 s when the expanding nucleation zone reaches one end of the fault, and dynamic slip appears to initiate at the other end of the fault when the expanding nucleation zone reaches the other end of the fault at approximately 0.2625 s. Once fault rupture is initiated, the rupture traverses the fault at approximately 2.5 km/s, slower than the shear wave speed of the sample, ***v_s_*** = 3.0 km/s [67], and typically reflects back along the length of the fault one or more times before the energy dissipates. As dynamic slip initiates, rapid fluctuations in the strain gage pair shear stress, AMR fault slip, and ultrasonic AE records can be seen propagating the length of the fault, and reflecting at the opposite end of the fault. These high frequency fluctuations are consistent with slip-pulses previously reported from the same test apparatus [13].

### 3.3. Spectral Analysis of Sensor Signals

**Figure 12:**
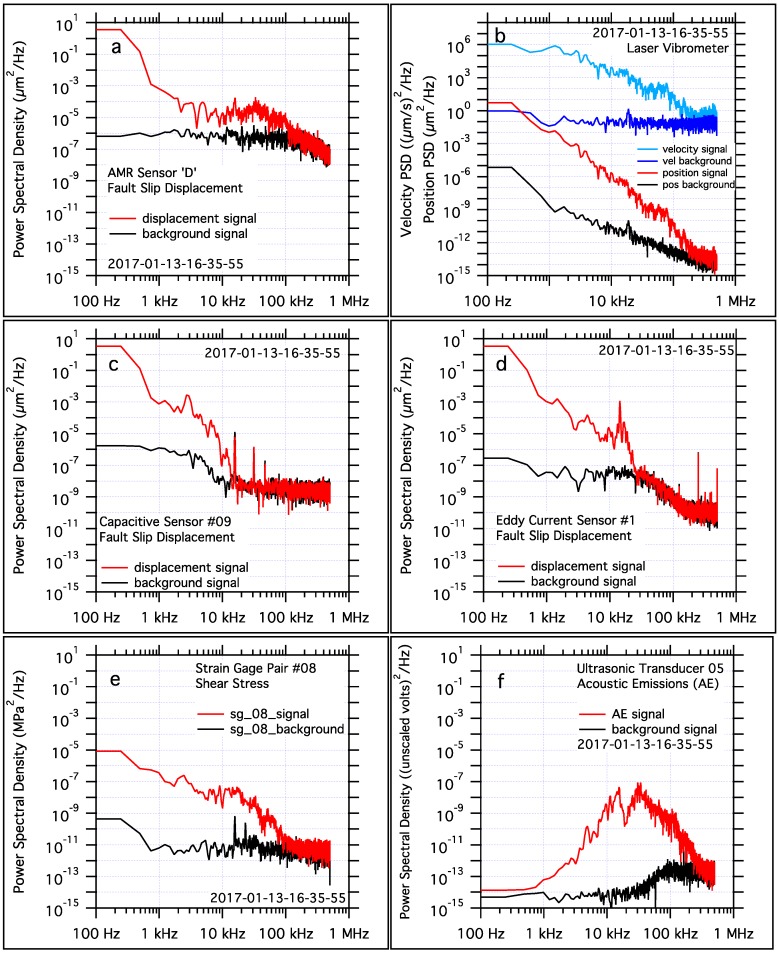
Power Spectral Density (PSD) analysis of signals collected by all of the sensors used in this study. Comparisons between each sensor signal and background spectra highlight the frequency response of each sensor. Frequency thresholds of interest include; 244 Hz is the lower bound of the PSD sensitivity, 400 Hz is approximately the (event duration (s))^−1^ of stick-slip in these tests, 1.2 kHz is approximately the (rupture propagation transit time)^−1^. Each plot is scaled identically to facilitate comparisons, with the exception of which has an enlarged y-axis to accommodate both laser fault slip and laser fault velocity PSD spectra. Spectra of signal and background noise from; one AMR fault slip displacement sensor (**a**), laser vibrometers measuring fault slip displacement and velocity (**b**), one capacitive sensor measuring fault slip displacement (**c**), one eddy current sensor measuring fault slip displacement (**d**), one pair of semiconductor strain gages measuring shear stress (**e**), one ultrasonic transducer measuring acoustic emissions emanating from the fault (**f**).

The frequency content of the sensor signals used in these tests was examined using Power Spectral Density (PSD) analysis. The same PSD analysis was applied to data from each sensor type; 8500 sample waveforms collected at 1 MS/s, a 4096 sample Hanning window, and the DC component of the sample waveform was removed. Each 8500-sample waveform included approximately equal amounts of pre-slip, dynamic slip, and post-slip data. The 8500-sample waveform length and the 4096 Hanning window dimension were also chosen so the PSD analysis would include frequencies below the approximately 0.002 s to 0.003 s event duration, so each PSD spectra might better represent the signal of the entire stick-slip process, not just the higher frequency dynamic slip aspects of fault slip. A second 8500-sample length segment of data from each sensor type, which significantly precedes the dynamic slip, was also subjected to the same PSD analysis to provide a background signal or noise spectra for comparison. Results of those analysis are presented in Figure 12. The PSD spectra for the AMR sensor fault slip displacement shows that these sensors as deployed have a response to about 100-kHz where the signal and background spectra merge. The PSD spectra for the laser vibrometer fault slip displacement reveals a signal response to about 200-kHz, though with less over all power but a higher signal to noise ratio when compared to the AMR sensors. The capacitive fault slip displacement signals appear to show signal to about 20-kHz, but the signal and noise spectra of that sensor both decline significantly above 3-kHz, as expected. The spectra for the eddy current fault slip displacement signal appears to decline significantly at about 20-kHz, well below the 50-kHz specified performance of that sensor. The shear stress spectra reveal signal response to about 100-kHz, which likely reflects the performance of the signal conditioning used with those semiconductor strain gage pairs, rather than the strain gages themselves which have a frequency response well beyond the ability of this lab to quantify. Finally, while the spectra for the ultrasonic transducer is shown for completeness, that sensors response is designed to be centered around 1 MHz, which is also the sampling rate for data analyzed in these tests. That the sensors expected peak response frequency is also the sampling rate for the data used for PSD analysis is an unfortunate coincidence.

**Figure 13:**
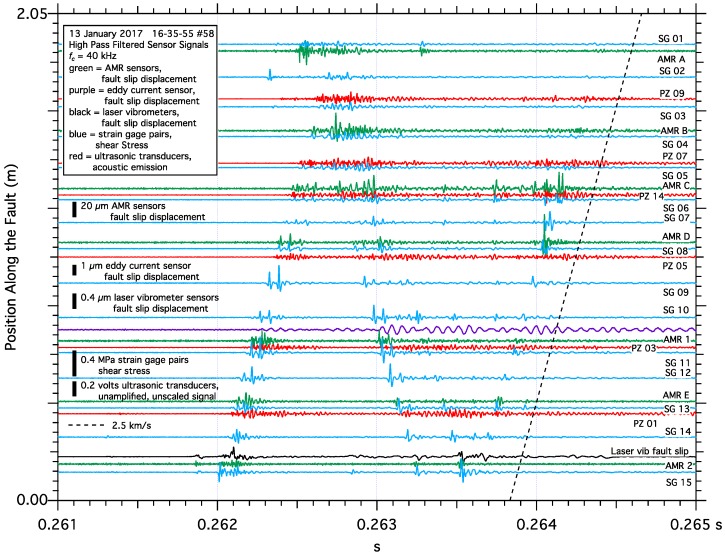
Sensor data that has been passed through a 40-kHz high pass filter to highlight its higher frequency content. Only sensors with signal content at or above 40-kHz are included on the plot, and signals are plotted along the y-axis as they are deployed along the fault trace to facilitate spatial and temporal comparison. Note that the laser vibrometer fault slip signal does contain high frequency oscillations, though with significantly smaller amplitudes when compared to the AMR fault slip sensor signals.

To better illustrate the high frequency fluctuations seen in the shear stress, AMR fault slip and ultrasonic AE records, all of the data records from the dynamic slip event shown in Figure 11 were passed through a 40-kHz high pass filter to highlight the higher frequency content of each signal, Figure 13. A detailed analysis of each sensors spectral content to identify an optimum high pass filter cut off frequency was beyond the scope of this report; however, 40-kHz appears to be an effective threshold. High pass filtered sensor signals are plotted such that their position along the *y*-axis of the plot corresponds to their position along the fault, and reveal clear patterns of energy traversing the length of the fault at approximately 2.5 km/s, likely coinciding with a passing rupture front. It is important to note that the scales for each sensor vary considerably. In particular, note that while the laser vibrometer fault slip displacement records seen in Figure 6, Figure 7, Figure 8 and Figure 9 do not appear to show any high frequency content, the PSD analysis of those records, Figure 12, shows that the laser vibrometer fault slip displacement records do contain signal at frequencies beyond 40-kHz. When passed through a high pass filter, high frequency oscillations in the laser vibrometer fault slip displacement record become visible and appears to correlate with other nearby sensors. The amplitude of the high frequency component of the vibrometer fault slip displacement record is only a fraction of a micron, and about 40× to 50× less than the amplitude of the high frequency fault slip displacement oscillations detected by the AMR sensor. The vibrometers were tracking the motion of that AMR sensor directly and in principal, the vibrometer and AMR position records should match. At lower frequencies, the vibrometer and AMR fault slip displacement records show good agreement, at higher frequencies (≥40 kHz) they show differences approaching two orders of magnitude in signal amplitude. The large difference in the amplitudes of the high frequency (≥40 kHz) components of the laser vibrometer and AMR sensor fault slip displacement records appears to be consistent with the approximately 40 dB difference in power between the AMR sensor and laser vibrometer fault slip displacement spectra at 40-kHz.

### 3.4. Electromagnetic Radiation

**Figure 14:**
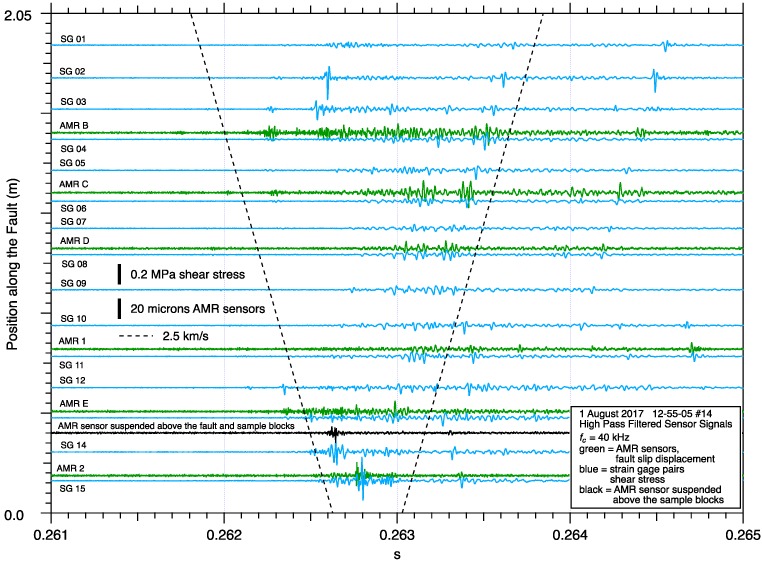
High pass filtered sensor signals during a test where a test AMR sensor was deployed suspended a few millimeters above the fault with no physical contact with the granite samples, but otherwise an identical deployment compared to the other AMR fault slip sensors. That suspended AMR sensor, the black trace in this figure, shows signal that cannot be caused by relative motion of that AMR sensor/magnet pair due to its physical isolation from the sample blocks.

**Figure 15:**
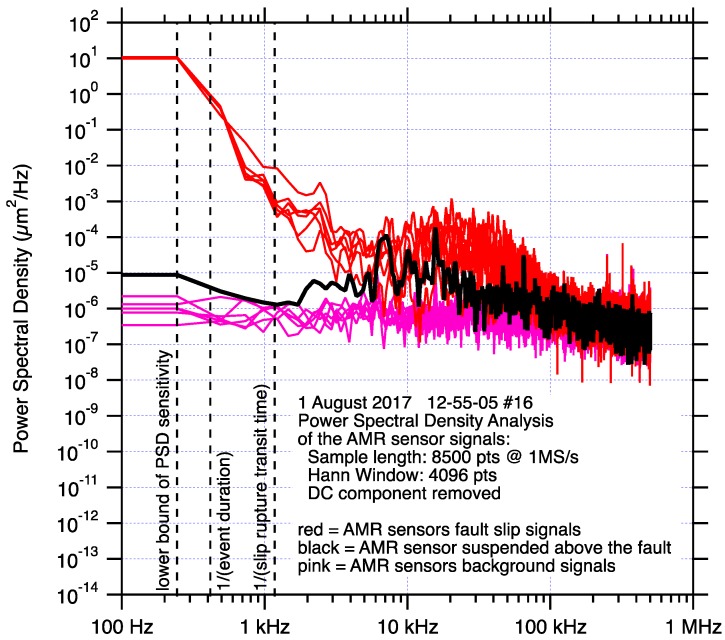
Comparison of the PSD spectra of AMR fault slip sensors deployed along the fault trace on the granite sample blocks, to the AMR sensor that was suspended a few mm above the fault trace. The suspended AMR sensor appears to have signal content above the background signals of all the AMR sensors, and within the low range of power amplitude of the AMR sensors deployed on the granite sample blocks measuring fault slip. Frequency thresholds are included for reference.

To investigate the source of the high frequency oscillations observed by the AMR sensors, an AMR sensor and target magnet were attached to single piece of prototype board which was deployed at different locations along the fault and signal was recorded during stick-slip events. Since neither the sensor nor the magnet had any physical contact with sample motion, there should be no differential motion between the sensor and the magnet resulting in a fixed/constant signal output. However, when this sensor was deployed both resting on, and later suspended above the fault to eliminate any contact with the samples, high frequency signal during stick-slip was detected. Figure 14 shows an example of high pass filtered slip and shear stress data from an event with a more complex dynamic slip pattern than from the event shown in Figure 13. Regardless of the complexity of the slip pattern, the AMR sensor suspended above the fault shows high frequency signal of roughly the same amplitude and coincident with other nearby AMR sensors which are responding to a passing dynamic fault rupture. PSD analysis of the data from several AMR sensors glued to the samples and the AMR sensor suspended above the fault, Figure 15, reveal that the suspended sensor has a signal spectra that is above the background noise level of those sensors. Since the source of that signal cannot be fault slip, and considering that the sensor is sensitive to magnetic fields, the source of the high frequency signal that the suspended AMR sensor is detecting, is possibly an EM signal related to the passing fault rupture.

## 4. Discussion

The AMR sensor described in this report is capable of acquiring detailed, accurate, and resonance free wideband measurements of dynamic fault slip displacement. Benchtop calibrations and scaling procedures for the AMR sensor described in this report can produce calibration curves with scaling errors within or below the performance specifications of other commonly used position sensors. The ability of the AMR sensor to accurately track rapid stick-slip fault motion and make accurate measurements of dynamic fault slip displacement in the lab was independently verified by the use of a pair of laser vibrometers. The laser vibrometers measured the differential fault slip velocity of the AMR sensor and its matching magnet directly, which was then integrated to fault slip displacement for a direct comparison to the AMR sensor signal. Side by side comparison of AMR sensor fault slip displacement signals to signals generated by close by capacitive and eddy current fault slip displacement sensors, also verify the performance of the AMR sensor with respect to its ability to accurately measure fault slip displacement.

The small AMR sensor package developed for these tests is a rigid, rugged, low-profile, easy to build device which facilitates its use in a variety of space sensitive scenarios. Observations in these tests show that the rigid sensor package eliminates the negative effects of sensor mount resonance, improving the quality of stick-slip fault slip data. The custom signal conditioning developed for these tests permits the sensor to be permanently bonded to the samples, and still allow for high resolution measurements while accommodating accumulating fault slip without manual re-positioning of the sensor.

The wideband response of the AMR sensor is verified by direct comparison to simultaneous laser vibrometer measurements and PSD analysis of the fault slip displacement records generated by those two sensing technologies. PSD analysis, Figure 12, shows that the sensor is sensitive to motion at frequencies to 100 kHz, which is comparable to the frequency response of the laser vibrometer fault slip displacement data. The discrepancy in amplitude of the high frequency signals detected by the AMR fault slip sensor vs the laser vibrometer fault slip displacement record is consistent with the difference in amplitude of the power spectra of those two sensors. The cause for the fault slip displacement amplitude discrepancy between the two sensors is unresolved at this time, though may be caused by the apparent sensitivity of the AMR sensor to fault generated EM signals, discussed later.

The small amount of fault slip detected by the AMR sensor after dynamic fault slip has ended, Figure 6a, could be apparent slip caused by the frame of reference of the laser vibrometers. Fault motion in these tests is left lateral, which may facilitate a counter-clockwise rotation of the sample blocks. The laser vibrometers measure the motion of the AMR sensor and its target magnet relative to the floor in the lab. In contrast, the AMR sensors measure fault slip directly, referenced to the sample blocks. Counter-clockwise sample block rotation following stick-slip fault slip motion, could cause the vibrometers to register block rotation of the sensor and the target as fault motion, resulting in additional, but apparent, fault slip. Apparent slip caused by block rotation should produce the largest signals at the corners of the sample blocks, the ends of the fault, presumably locations of maximum rotation motion. Measurements of fault slip near the middle of the fault in the center of the sample blocks, shows no post stick-slip fault motion, Figure 8a, presumably where block rotation effects would be minimized. The test apparatus resonates at approximately 425 Hz after each stick-slip event, evidenced by audible ringing and oscillations in the shear strain gage pair records, and may facilitate post slip rotation or other motion of the sample blocks. Additional measurements of the motion of the sample blocks relative to themselves, the loading frame, and the floor of the lab would be required to positively identify the source of the apparent fault slip observed in Figure 6a.

The appearance of the high frequency fluctuations in the AMR dynamic slip records present an unanticipated, and interesting set of observations. These signals appear to be related to the high frequency, low amplitude slip pulses traveling the length of the fault as a part of the fault rupture process [13]. If the high frequency oscillations in the AMR records represent traveling slip pulses, then perhaps they represent actual deformation near the fault. However, the amplitude of those high frequency signals in the AMR fault slip records is quite large compared to the total amount of fault slip. Fault slip displacement signals in Figure 6a and Figure 8a, as well as high pass filtered fault slip displacement data in Figure 13 and Figure 14, suggest that the magnitude of the high frequency fault slip signals is tens of microns of fault slip motion. Considering that total fault slip in these events was about 130 to 140 microns, the amplitude of the high frequency signals represents a significant amount of forward, and highly unlikely backward, fault motion during strike-slip fault block motion. If the high frequency fluctuations in the AMR records represent actual fault slip, with tens of microns of positive and negative fault motion, then the velocity record should show matching high frequency oscillations reflecting positive and negative velocity, but they do not. However, the laser vibrometer differential fault slip velocity signals resulting from the simultaneous tracking an AMR sensor and magnet, then integrated to fault slip displacement, do show high frequency fault motions, though with amplitudes of only a fraction of a micron, about two orders of magnitude less than the same motion detected by the AMR sensors.

If the high frequency fault slip displacement oscillations detected by the AMR sensors represent fault motion or deformation, then the coincident high frequency oscillations seen in the shear stress records could scale with that motion. Using the shear modulus of the sample rock, as well as the change in shear stress observed by the shear strain gage pairs, we can use the relation

***G*** = *τ**_xy_***/*γ**_xy_*** = (**F**/**A**)/(**∆*x***/***l***) (1)

where ***G*** = the shear modulus of the sample, *τ**_xy_*** and ***F***/***A*** = shear stress, *γ**_xy_*** and **∆*x***/***l*** = shear strain, to roughly estimate the motion that might be expected for a given magnitude of shear stress fluctuation. The shear modulus for Sierra White granite is about 25 GPa [67,69], and the high pass filtered signals in Figure 13 show shear stress oscillations with amplitudes of about 0.25 MPa. The shear stress is measured over a distance ***l*** of about 1 cm on the sample surface, the approximate length dimension of the strain gage pair. Solving for **∆*x***, 0.1 microns is the approximate amplitude of shear deformation that would accompany the shear stress oscillations of the magnitude observed in these tests. While this number is consistent with the amplitude of high frequency fault slip displacement oscillations detected by the laser vibrometers, it is substantially less than the amplitude of apparent high frequency fault slip displacement oscillations measured by the AMR sensors. A traveling elastic wave related to a travelling fault rupture front would be consistent with temporal appearance of these high frequency oscillations, and the small amplitude of these oscillations determined by the laser vibrometer appears to be consistent with the modulus of the sample material and the amplitude of shear stress fluctuations related to the passing rupture front.

One possible mechanism for the high frequency signal oscillations seen in the AMR fault slip records is the generation of EM signals related to fault rupture or fault rupture propagation. The AMR sensor is a magnetic sensor, and may be sensitive to EM signals generated by the stick-slip fault motions generated in these tests. It is important to note that these tests were not designed to look for EM signals, and as such, appropriate EM sensing equipment was not deployed. The possibility that EM signals could be generated under these test conditions is not a surprise. Various mechanisms have been invoked for the generation of earthquake and volcanic EM signals [21,22,23,24,25], including piezomagnetic and electrokinetic effects, elastic waves propagating through the Earth’s crust, and others, have been associated with the generation of EM signals. The lack of ferro-crystalline minerals or fluid saturated pore space in the granite used in these tests disqualifies both piezoresistive and electokinetic mechanisms in these tests. A propagating elastic rupture front generating triboelectric EM signals related to microcracking and rock shearing at the rupture front as the rupture front passes [23], is a plausible mechanism for the generation of EM signals in this test configuration. In addition, these granite samples are quartz rich, which could also facilitate the generation of transient piezoelectric charge which the AMR sensors could be sensitive to.

Why some sensors deployed in these tests detect the apparent EM signals and some don’t, pose some interesting questions. For example, the AMR sensor is designed to be sensitive to magnetic fields. However, it was operated in a ‘saturating’ magnetic field of approximately 80 Gauss to in part, reduce the effects of stray magnetic fields, which might suggest that EM signals generated by faulting could be of a sufficient magnitude to be readily detectable by other means. The strain gage pairs, as well as the eddy current and capacitive position sensors all generate signal fluctuations when a hand-held magnet is passed close by those sensors, suggesting that their signals may all be influenced by transient EM signals. The semiconductor strain gages themselves apparently show no magnetostriction and very little magnetoresistive effect [70], though their lead wirers could act as small antenna receptive to EM signals. Indeed, lead wire weaving techniques and non-inductive foil strain gages exist that are designed specifically to minimize noise pick-up from EM signals. The use of non-inductive strain gages and better shielded lead wires in future tests could help better identify the source of higher frequency oscillations observed in the strain gage pairs in these tests. The use of AC excitation (vs. DC excitation as in these tests) with the existing strain gage pairs to reduce EM sensitivity, or the deployment of optical strain gages which are insensitive to EM signals, may offer insights, though these technologies are both bandwidth limited and best suited for quasi-static strain measurements. The relatively low bandwidth of the capacitive position sensors appears to render them incapable of generating signals related to any higher frequency EM signals. The eddy current sensors which should have a 50-kHz signal bandwidth, and appear to be sensitive to a close by magnet, do not detect these apparent EM signals. The PSD spectral analysis of the eddy current sensors suggest that their actual frequency response is much less than 50-kHz, possibly rendering then insensitive to EM signals generated during these tests. The ultrasonic transducers did not appear to be sensitive to the presence of a close by magnet and suggests that those sensors are only responding to ultrasonic acoustic emissions generated during dynamic rupture. Testing currently beyond the capabilities of this lab would be required to accurately characterize the nature of any EM emissions generated in this testing apparatus, and its effect on the sensors used in these tests.

The stick-slip data collected using the AMR sensor/magnet pair that was suspended above the sample blocks (Figure 14), show high frequency oscillating signals comparable to AMR sensor/magnet pairs that were attached to the sample blocks during stick-slip events. Spectral analysis of the suspended AMR sensor vs glued down AMR sensors, Figure 15, also shows that the two signals are comparable, though the suspended sensors spectral amplitude is a bit less than that of the glued down sensors, it is still above the noise level of both sensors and suggests that at least some of the high frequency AMR fault slip displacement signal is derived from possible EM emissions from the sample blocks during dynamic stick-slip motion. High pass filtered laser vibrometer fault slip displacement data detect the high frequency motion of the glued down AMR sensors, but about two orders of magnitude less than what the AMR sensors detect, suggests that the while AMR sensors are clearly responding accurately to fault slip displacement, they also have some sensitivity to possible EM signals related to fault slip processes.

To fully understand the source physics of EM signals observed in tests like these, appropriate sensors, experimental techniques, and shielding from extraneous sources of EM energy are all required. Future experiments should employ sensors to quantify any quasi-static or wideband transient EM signals that may be generated during the generation of small strike-slip earthquakes in the lab. The use of sensors insensitive to EM signals to measure fault slip, fault slip velocity, strain patterns and accelerations along the fault, would all contribute to the understanding of the source of the apparent EM signals reported here. Specific to the AMR sensor, using more powerful target magnets may improve the AMR sensors ability to reject extraneous signals, though, how stronger magnets might adversely affect other close by sensors would need to be considered. Small scale tests using samples with varying amounts of quartz and silica, and similarly varying piezoelectric effects, may be insightful as well. While granite is one of the more silica rich igneous rock types, experiments using diorite, gabbro or peridotite with progressively less silica content, may provide helpful insights into coseismic EM emissions.

## 5. Conclusions

The AMR magnetoresistive sensor as deployed in these tests has been shown to be a simple, and rugged sensor that could facilitate the acquisition of wideband, accurate, and resonance free measurements of dynamic stick-slip fault motions in laboratory geophysical experiments. The small size facilitates its use on smaller samples or permits the installation of additional sensors on larger well instrumented samples. Signal conditioning electronics permit the sensor to be used at gains high enough to capture fine fault slip motion detail, in addition to accommodating accumulating fault slip without repositioning the sensor. Simple spectral analysis techniques revealed significant insights into the performance of each sensor used in these tests. The observations of what appear to be EM emissions related to fault rupture propagation detected by this sensor, could, with appropriate sensors and experimental techniques, offer insights into the source physics of EM emissions, and their relationship to earthquake physics.

## Figures and Tables

**Figure A1:**
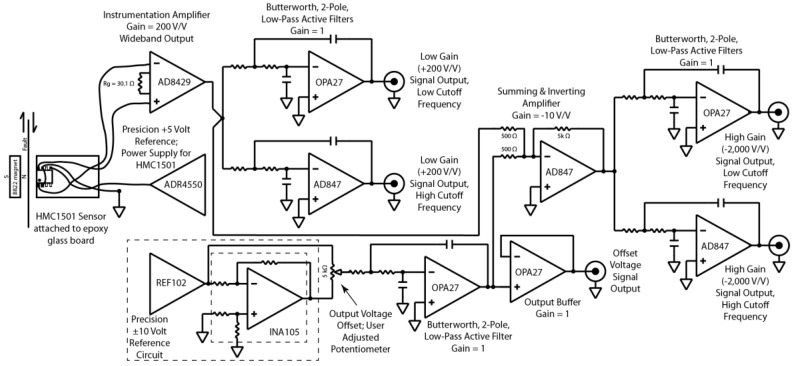
Diagram of the wide-band circuit built to amplify and offset the output signal of the AMR sensor used these tests. The AMR sensor and its target magnet are shown as deployed on the sample blocks relative to the strike-slip fault, and as connected to the circuit.

## Appendix A

(1) Signal conditioning:

The signal conditioning used to excite, and amplify the signal generated by the AMR sensor needs to accomplish two tasks. One task is to amplify the signal to a sufficient level such that high resolution digital signals can be obtained. The other task is to electronically accommodate accumulating slip along the fault after each stick-slip event, since the sensor and magnet are permanently attached to the samples, and not adjustable themselves. To accomplish this, a two-gain stage amplifier is developed, with the capability for output offset incorporated into the design (Appendix Figure A1).

To power the AMR sensor, a low noise precision +5 V reference voltage (Analog Devices ADR4550) is employed to provide the excitation voltage directly to the AMR sensor. The input bridge resistance of AMR (4 kΩ minimum) is high enough that the current draw on the ADR4550 voltage reference is within its specifications, eliminates the need for a current buffer amplifier to provide adequate power to the sensor, and simplifies the circuit design.

The first amplifier stage employs a low noise wideband instrumentation amplifier (Analog Devices AD8429) with a user defined gain of +200 V/V. At that gain, the output of the AMR sensor, with a 5 mm gap between it and the magnet, can accommodate approximately 10 mm of lateral motion (fault slip) of the magnet while keeping the amplified voltage output swing between ±10 V. The coarse resolution of that signal, about 500 µm/volt, is not ideal for making precision position measurements, however, it is convenient for calibrating the sensor over its entire sensing range. The second amplifier stage sums the signal from the instrumentation amplifier with a user adjustable, ±10 volt offset, and amplifies the summed signal using a wideband op-amp (Analog Devices AD847) configured with a user defined gain of −10 V/V. The offset voltage adjustment permits the easy biasing of the instrumentation amplifier output so the higher gain signal output remains on-scale (within ±10 volts) as slip accumulates during successive tests. The additional gain improves sensor resolution to about 50 µm/volt, comparable to the sensitivity of the existing sensors used for these measurements.

The ±10 volt voltage offset is designed to be both stable, and low noise. The offset signal is generated by a circuit employing a +10 volt precision voltage reference (Texas Instruments REF102), a precision differential amplifier (Texas Instruments INA105), a 10-turn wirewound precision potentiometer, and is buffered and filtered through a low pass active filter circuit built around a low noise Texas Instruments OPA27 op-amp.

The output of the first amplifier stage is passed on to the second amplifier stage unfiltered. The output of first amplifier stage is also made available for user monitoring and (or) recording using two user configurable Butterworth low pass active filter/buffer circuits. One active filter/buffer circuit is configured with a cutoff frequency appropriate for slower long term data recording rates. The other active filter/buffer circuit is configured with no filtering or as a low pass filter with a cutoff frequency appropriate for wideband transient signal recording. The output of the second amplifier stage is also made available for user monitoring and/or data recording using an identical pair of low pass filter/buffer circuits. A buffered output of the offset voltage is also made available for monitoring or recording.

Details for circuit layout best practices, and specifications for the resistors, capacitors and other components incorporated into this circuit are detailed within the data sheets for each integrated circuit identified above. The circuit boards employed in these tests were handmade using off the shelf epoxy glass prototyping board, DIP sockets, connectors, potentiometers, enclosures, etc. Metal film resistors are employed throughout, and a low temperature coefficient spec is identified for all gain resistors. Details of the op-amps, resistors, and capacitors used to set the response characteristics of each individual active filter can be quickly identified for specific test requirements using several online calculators, for example; the Analog Filter Wizard from Analog Devices [71] or the WEBENCH^®^ Filter Designer from Texas Instruments [72].

(2) Calibration:

The gain of the first instrumentation amplifier in the signal conditioning circuit is set so that the entire sensing range of the sensor, about 10 mm, produces a full-scale voltage output swing of about ±10 volts, so the entire range of motion can be documented. Scaling the sensor signal from either the first or second gain stage using a linear scale is a simple matter. Scaling the sensor signal from the first gain stage using a polynomial calibration is also a simple matter. However, if a polynomial calibration curve is to be applied to the higher resolution second gain stage output, to better accommodate the sinusoidal output of the AMR sensor, a procedure is needed so the polynomial scaling is correctly applied. A polynomial scale is valid only when applied to the same position/output data pairs from which it was derived.

Scaling sensor signals from the second gain stage of the signal conditioner is a multistep process. Step 1: both the lower first gain, and the higher second gain sensor signals are recorded. Step 2: the higher gain signal is divided by the gain of the second stage amplifier, −10 in this case. The resulting signal should match in amplitude and sense the output of the first gain stage, but with an offset. That offset value should be a constant value, and equal to the user adjustable offset voltage summed in the second gain stage of the signal conditioning circuit. Step 3: subtract the offset from the divided high gain signal. The scaled down and offset adjusted high gain signal output should now match the low gain signal in both amplitude and sense, though the high gain signal will show significantly more detail in the signal. Step 4: a polynomial calibration (or any other calibration curve) determined from the low gain bench top calibration, can be now applied to the high gain signal to obtain a scaled displacement record. It is critical that if a polynomial, or any other nonlinear calibration is applied to the data, that the calibration curve is applied such that the recorded voltage that is converted to microns is the same range as was used to determine the curve fit. This is particularly relevant in this study where different short segment calibration curves are applied to the recorded data depending on the amount of accumulated fault slip.

## References

[1] Dieterich J.H. (1979). Modeling of Rock Friction 1. Experimental Results and Constitutive Equations. J. Geophys. Res..

[2] Dieterich J.H., Carter N.L., Friedman M., Logan J.M., Stearns D.W. (1981). Constitutive properties of faults with simulated gouge. Mechanical Behavior of Crustal Rocks.

[3] Ruina A.L. (1983). Slip Instability and State Variable Friction Laws. J. Geophys. Res..

[4] Tullis T.E., Weeks J.D. (1986). Constitutive Behavior and Stability of Frictional Sliding of Granite. PAGEOPH.

[5] Blanpied M.L., Tullis T.E., Weeks J.D. (1987). Frictional behavior of granite at low and high sliding velocities. Geophys. Res. Lett..

[6] Linker M.F., Dietrich J.H. (1992). Effects of variable normal stress on rock friction: Observations and constitutive equations. J. Geophys. Res..

[7] Kilgore B.D., Blanpied M.L., Dietrich J.H. (1993). Velocity-dependent Friction of Granite over a Wide Range of Conditions. Geophys. Res. Lett..

[8] Marone C., Kilgore B. (1993). Scaling the critical slip distance for seismic faulting with shear strain in fault zones. Nature.

[9] Kilgore B., Lozos J., Beeler N., Oglesby D. (2012). Laboratory observations of fault strength in response to changes in normal stress. J. Appl. Mech..

[10] Beeler N., Kilgore B., McGarr A., Fletcher J., Evans J., Baker S.R. (2012). Observed source parameters for dynamic rupture with non-uniform initial stress and relatively high fracture energy. J. Struct. Geol..

[11] McGarr A. (2012). Relating stick-slip friction experiments to earthquake source parameters. Geophys. Res. Lett..

[12] McLaskey G.C., Kilgore B.D., Lockner D.A., Beeler N.M. (2014). Laboratory generated M-6 earthquakes. PAGEOPH.

[13] McLaskey G.C., Kilgore B.D., Beeler N.M. (2015). Slip-pulse rupture behavior on a 2 m granite fault. Geophys. Res. Lett..

[14] Selvadurai P.A., Glaser S.D. (2015). Laboratory-developed contact models controlling instability on frictional faults. J. Geophys. Res..

[15] Togo T., Shimamoto T., Yamashita F., Fukuyama E., Mizoguchi K., Urata Y. (2015). Stick-slip behavior of Indian gabbro as studied using a NIED large-scale biaxial friction apparatus. Earthq. Sci..

[16] Urata Y., Yamashita F., Fukuyama E., Noda H., Mizoguchi K. (2016). Apparent dependence of rate- and state-dependent friction parameters on loading velocity and cumulative displacement inferred from large-scale biaxial fiction experiments. PAGEOPH.

[17] Passelegue F.X., Schubnel A., Nielsen S., Bhat H.S., Deldicque D., Madariaga R. (2016). Dynamic rupture processes inferred from laboratory microearthquakes. J. Geophys. Res..

[18] Lockner D., Kilgore B., Beeler N., Moore D., Thomas M., Bhat H.S., Mitchell T. (2017). The transition from frictional sliding to shear melting in laboratory stick-slip experiments. Fault Zone Dynamic Processes: Evolution of Fault Properties during Seismic Rupture.

[19] Kilgore B., McGarr A., Beeler N., Lockner D., Thomas M., Bhat H.S., Mitchell T. (2017). Earthquake source properties from instrumented stick-slip. Fault Zone Dynamic Processes: Evolution of Fault Properties during Seismic Rupture.

[20] Ohnaka M., Kuwahara Y., Yamamoto K. (1987). Constitutive relations between dynamic physical parameters near a tip of the propagating slip zone during stick-slip shear failure. Tectonophysics.

[21] Johnston M.J.S., Mueller R.J., Sasai Y. (1994). Magnetic field observations in the near-field the 28 June 1992 M_w_ 7.3 Landers, California, earthquake. Bull. Seismol. Soc. Am..

[22] Johnston M.J.S. (1997). Review of Electrical and Magnetic Fields Accompanying Seismic and Volcanic Activity. Surv. Geophys..

[23] Johnston M.J.S., Lee W.H.K., Kanamori H., Jennings P.C., Kisslinger C. (2002). Electromagnetic fields generated by earthquakes. International Handbook of Earthquake and Engineering Seismology.

[24] Johnston M.J.S., Borcherdt R.D., Linde A.T., Gladwin M.T. (2006). Continuous Borehole Strain and Pore Pressure in the Near-Field of the September 28, 2004 Parkfield Earthquake: Implications for Nucleation, Fault Response, Earthquake Prediction and Tremor. Bull. Seis. Soc. Am..

[25] Surkov V.V., Pilipenko V.A. (1997). Magnetic effects due to earthquakes and underground explosions: A review. Ann. Geophys..

[26] Thomas J.N., Love J.J., Johnston M.J.S. (2009). On the reported magnetic precursor of the 1989 Loma Prieta earthquakes. Phys. Earth Planet. Inter..

[27] Thomas J.N., Love J.J., Johnston M.J.S., Yumoto K. (2009). On the reported magnetic precursor of the 1993 Guam earthquake. Geophys. Res. Lett..

[28] Masci F. (2012). Comment on “Possible association between anomalous geomagnetic variations and the Molise earthquakes at central Italy during 2002” by Takla et al. (2011). Phys. Earth Planet. Inter..

[29] Masci F. (2012). On the ULF magnetic ratio increase before the 2008 Iwate–Miyagi Nairiku earthquake by Hirano and Hattori (2011). J. Asian Earth Sci..

[30] Masci F. (2012). The study of ionospheric anomalies in Japan area during 1998–2010 by Kon et al.: An inaccurate claim of earthquake-related signatures?. J. Asian Earth Sci..

[31] Masci F. (2013). Brief communication “Further comments on the ionospheric precursor of the 1999 Hector Mine earthquake”. Nat. Hazards Earth Syst. Sci..

[32] Masci F. (2013). On the multi-fractal characteristics of the ULF geomagnetic field before the 1993 Guam earthquake. Nat. Hazards Earth Syst. Sci..

[33] Masci F., Thomas J.N. (2014). Comment on “Temporal and spatial precursors in ionospheric total electron content of the 16 October 1999 Mw7.1 Hector Mine earthquake”, by Su et al. (2013). J. Geophys. Res. Space Phys..

[34] Masci F., Thomas J.N. (2015). On the reliability of the Spatial Scintillation Index to identify ionospheric precursors of earthquakes. Radio Sci..

[35] Masci F., Thomas J.N., Villani F., Secan J.A., Rivera N. (2015). On the onset of ionospheric precursors 40 minutes before strong earthquakes. J. Geophys. Res. Space Phys..

[36] Nitsan U. (1977). Electromagnetic emission accompanying fracture of quartz-bearing rocks. Geophys. Res. Lett..

[37] Warwick J.W., Stoker C., Meyer T.R. (1982). Radio emission associated with rock fracture: Possible application to the Great Chilean Earthquake of May 22, 1960. J. Geophs. Res..

[38] Ogawa T., Oike K., Miura T. (1985). Electromagnetic radiations from rocks. J. Geophys. Res..

[39] Cress G.O., Brady B.T., Rowell G.A. (1987). Sources of electromagnetic radiation from fracture of rock samples in the laboratory. Geophys. Res. Lett..

[40] Yamada I., Masuda K., Mizutani H. (1989). Electromagnetic and acoustic emission associated with rock fracture. Phys. Earth Planet. Inter..

[41] Enomoto Y., Hashimoto H. (1990). Emission of charged particles from indentation fracture of rocks. Nature.

[42] Yoshida S., Clint O., Sammonds P.R. (1998). Electric potential changes prior to shear fracture in dry and saturated rocks. Geophys. Res. Lett..

[43] Rabinovitch A., Frid V., Bahat D., Goldbaum J. (2000). Fracture area calculation from electromagnetic radiation and its use in chalk failure analysis. Int. J. Rock Mech. Min. Sci..

[44] Yoshida S., Ogawa T. (2004). Electromagnetic emissions from dry and wet granite associated with acoustic emissions. J. Geophys. Res..

[45] Mori Y., Obata Y., Sikula J. (2009). Acoustic and electromagnetic emission from crack created in rock sample under deformation. J. Acoust. Emiss..

[46] Zhu T., Zhou J., Wang H. (2013). Electromagnetic emissions during dilating fracture of a rock. J. Asian Earth Sci..

[47] Lockner D.A., Byerlee J.D., Kuksenko V.S., Ponomarev A.V. (1986). Stick slip, charge separation and decay. PAGEOPH.

[48] Yoshida S., Uyeshima M., Nakatani M. (1997). Electric potential changes associated with slip failure of granite: Preseismic and coseismic signals. J. Geophys. Res..

[49] Rabinovitch A., Shay A., Liraz R., Frid V., Bahat D. (2005). Electromagnetic radiation emitted during friction process. Int. J. Fract..

[50] Tsutsumi A., Shirai N. (2008). Electromagnetic signals associated with stick–slip of quartz-free rocks. Tectonophysics.

[51] Onuma K., Muto J., Nagahama H., Otsuki K. (2011). Electric potential changes associated with nucleation of stick-slip of simulated gouges. Tectonophysics.

[52] Tsutsui M. (2014). Behaviors of electromagnetic waves directly excited by earthquakes. IEEE Geosci. Remote Sens. Lett..

[53] Hu H., Gao Y. (2011). Electromagnetic field generated by a finite fault due to electrokinetic effect. J. Geophys. Res..

[54] Gao Y., Chen X., Hu H., Wen J., Tang J., Fang G. (2014). Induced electromagnetic field by seismic waves in Earth’s magnetic field. J. Geophys. Res. Solid Earth.

[55] Bratland T., Hong W. Linear Position Sensing Using Magnetoresistive Sensors. https://scholar.google.com/scholar?q=%22Linear+Position+Sensing+Using+Magnetoresistive+Sensors%2C%22.

[56] Honeywell International Inc. Applications of Magnetic Position Sensors. https://aerocontent.honeywell.com/aero/common/documents/Applications-of-Magnetic-Position-Sensors.pdf.

[57] Honeywell International Inc. Magnetic Displacement Sensors HMC1501/1512. https://aerocontent.honeywell.com/aero/common/documents/myaerospacecatalog-documents/Missiles-Munitions/HMC1501-1512.pdf.

[58] McGuire T.R., Potter R.I. (1975). Anisotropic magnetoresistance in ferromagnetic 3D alloys. IEEE Trans. Magn..

[59] Nickle J. (1995). Magnetoresistance Overview. http://www.hpl.hp.com/techreports/95/HPL-95-60.html.

[60] Caruso M.J., Bratland T. (1999). Anisotropic magnetoresistive sensors theory and application. Sens. Mag..

[61] Sung G., Shalyguina E.E., Shin K.-H. (1999). Theoretical interpretation of positive magnetoresistance in Permalloy film. J. Magn..

[62] Finlay C.C., Maus S., Beggan C.D., Bondar T.N., Chambodut A., Chernova T.A., Chulliat A., Golovkov V.P., Hamilton B., Hamoudi M. (2010). International Geomagnetic Reference Field: The eleventh generation. Geophys. J. Int..

[63] K&J Magnetics, Inc.. www.kjmagnetics.com.

[64] AlphaLab Inc.. www.alphalabinc.com.

[65] Dieterich J.H. (1981). Potential for geophysical experiments in large scale tests. Geophys. Res. Lett..

[66] Okubo P.G., Dietrich J.H. (1981). Fracture energy of stick-slip events in a large scale biaxial experiment. Geophys. Res. Lett..

[67] Lockner D.A., Okubo P.G., Dieterich J.H. (1982). Containment of stick-slip failures on a simulated fault by pore fluid injection. Geophys. Res. Lett..

[68] Lockner D.A., Okubo P.G. (1983). Measurements of Frictional Heating in Granite. J. Geophys. Res..

[69] Okubo P.G., Dietrich J.H. (1984). Effects of physical fault properties on frictional instabilities produced on simulated faults. J. Geophys. Res..

[70] Kinnaman D. Kulite Semiconductor Products, Inc., Strain Gage Manual. https://www.kulite.com/docs/products_overview/StrainGageManualDigital.pdf.

[71] Analog Devices Analog Filter Wizard. http://www.analog.com/designtools/en/filterwizard/.

[72] Texas Instruments WEBENCH^®^ Filter Designer. http://www.ti.com/filterdesigner.

